# Supplementary material for: Biochemical Defense Responses in Red Rice Genotypes Possessing Differential Resistance to Brown Planthopper, Nilaparvata lugens (Stål)
Source: Insects. 2023 Jul 13;14(7):632. doi: 10.3390/insects14070632 (PMC10380536; doi:10.3390/insects14070632)
Supplement: Supplementary file 1 [file insects-14-00632-s001.zip › insects-2402867-supplementary.pdf]

Table S1: ANOVA of the interaction between red rice genotypes, defense enzymes and time series as influenced by *N. lugens* infestation

Source	DF	Sum of Squares	Mean Square	F-Ratio	p-Value	CD at 5%
Replication	2	11.053	5.527	3.220	0.0402	-
Genotypes (F1)	30	4607.778	153.593	89.499	<.0001	0.43
Defense Enzymes (F2)	5	162887.159	32577.432	18982.964	<.0001	0.19
Time series (F3)	3	6031.147	2010.382	1171.456	<.0001	0.15
Genotypes (F1) x Defense Enzymes (F2)	150	11842.081	78.947	46.003	<.0001	1.05
Defense Enzymes (F2) x Time series (F3)	15	25902.127	1726.809	1006.216	<.0001	0.86
Genotypes (F1) x Time Series (F3)	90	557.223	6.191	3.608	<.0001	0.38
Genotype (F1) x Defense Enzymes (F2) x Time series (F3)	450	3348.471	7.441	4.336	<.0001	28.40
Error	1486	2550.185	1.716	-	-	-
Total	2231	217737.223	.	-	-	-

F1 = Factor 1, F2 = Factor 2, F3 = Factor 3, CV% = 6.760, R^2 = 0.988

Table S2: Total Phenol (mg/g FW) accumulation in red rice genotypes after *N. lugens* feeding

Sl. No	Category (Score)	Genotypes	0 HAR	24 HAI	48 HAI	72 HAI
1	R (3)	Bavdi	14.35(22.26) *ab	33.96(35.64) ab	61.22(51.48)abc	48.62(44.21)a-d
2	R (3)	Bacharya Khuta	22.23(28.13)a	27.96(31.92)ab	63.15(52.62)a	51.71(45.98)a
3	HR (1)	Sonahanan	28.33(32.16)a	35.27(36.44)a	62.33(52.14)de	43.21(41.10)a-f
4	HR (1)	Mata Meher	29.86(33.12) a	33.76(35.53)ab	65.98(54.32)a	49.07(44.47)a-c
5	HR (1)	Hermonona	23.64(29.09)a	32.14(34.53)ab	64.34(53.33)a	45.35(42.33)a-e
6	HR (1)	Manipuri Black	23.69(29.12)a	35.62(36.64)a	62.05(51.97)ab	41.85(40.31)a-f
7	R (3)	Sati Kh-16	13.30(21.39)ab	27.10(31.37)ab	48.21(43.98)de	40.91(39.77)a-f
8	R (3)	Uttar Bangalocal-3	27.84(31.84)a	32.20(34.57)ab	46.45(42.96)de	36.83(37.36)c-f
9	R (3)	Bandi Marhan	15.54(23.22)ab	29.41(32.84)ab	45.39(42.36)de	37.90(38.00)b-f
10	R (3)	Chapa khusi-1	13.36(21.44) ab	32.78(34.93)ab	49.59(44.76)de	43.48(41.25)a-f
11	R (3)	Chadai Guda	12.20(20.44)ab	29.36(32.81)ab	44.82(42.03)de	38.97(38.63)a-f
12	R (3)	Manipuri	19.44(26.16)ab	30.66(33.62)ab	51.85(46.06)b-d	38.83(38.55)a-f
13	R (3)	Mujni	12.16(20.41)ab	32.69(34.87)ab	50.27(45.15)c-e	33.86(35.58)e-g
14	R (3)	Kakharua	18.21(25.26)ab	32.71(34.88)ab	48.76(44.29)de	39.56(38.97)a-f
15	R (3)	Lechade dhan	13.40(21.48)ab	32.14(34.53)ab	45.73(42.55)de	39.66(39.03)a-f
16	MR (5)	Rajesh	11.49(19.81)ab	28.49(32.26)ab	46.72(43.12)de	35.30(36.45)d-f
17	MR (5)	Meghi	13.69 (21.72) ab	29.18 (32.70)ab	46.57(43.03) de	39.27 (38.80)a-f
18	MR (5)	Balam	11.31(19.65) ab	21.58 (27.68) b	43.52 (41.28)de	37.97(38.04)b-f
19	MR (5)	Khuti Dhan	9.32(17.77)ab	30.91(33.78)ab	45.60(42.48)de	38.71(38.48)a-f
20	MR (5)	Gerua Mudi	10.14(18.57)ab	27.63(31.71)ab	43.57(41.30)de	35.62(36.64)d-f
21	MR (5)	Sakta-1	8.76 (17.21)ab	26.07(30.70)ab	42.23(40.53)d	22.18(28.10)g
22	MR (5)	Shial Bhomra	14.50(22.39)ab	25.60(30.40)ab	46.75(43.14)de	35.95(36.84)c-f
23	MR (5)	Binni	17.19(24.49)ab	28.70(32.39)ab	42.68(40.79)de	35.45(36.54)d-f

24	MR (5)	Kalshepa Kh-16	11.11(19.47)ab	25.42(30.28)ab	41.97(40.38)de	37.37(37.68)b-f
25	MR (5)	Maria Dhan-2	11.95(20.23)ab	29.04(32.61)ab	46.75(43.14)de	36.83(37.36)c-f
26	MR (5)	Maha Baisur	15.89(23.49)ab	28.74(32.42)ab	46.52(43.01)de	34.07(35.71)e-g
27	MR (5)	Mudi Futa	9.83(18.27)ab	30.67(33.63)ab	39.51(38.95)e	30.87(33.75)fg
28	MR (5)	Sarka Dhan	10.48(18.89)ab	27.64(31.72)ab	40.37(39.45)e	32.54(34.78)e-g
29	HR (1)	Salkati (Check)	27.23(31.46)a	32.56(34.79)ab	71.32(57.62)a	50.70(45.40)ab
30	HR (1)	PTB-33 (Check)	27.46(31.60)a	32.38(34.68)ab	62.57(52.28)ab	41.86(40.32)a-f
31	HS (9)	TN1 (Check)	4.55(12.31)b	6.80(15.11)c	9.39(17.84)f	5.57(13.66)h
DF			30	30	30	30
Treatment Sum of Squares			2592.04	1322.29	4562.33	2992.22
F value			4.2634	8.7692	48.3906	99.7409
p-Value			<.0001	<.0001	<.0001	<.0001
CV(%)			19.51	6.87	3.99	5.60
SE(d)			3.676	1.831	1.447	1.730
Tukey HSD at 1%			16.51	8.224	6.503	7.771

Mean of three replications in a column, followed by the common letter are not significantly different (P = 0.05)

*Figures in parenthesis are transformed square root values; HAI: Hour after infestation

HR: Highly Resistant, MR: Moderately Resistant, MS: Moderately Susceptible, HS: Highly Susceptible

Table S3: Peroxidase (POD) activity (UA/g FW) in red rice genotypes after *N. lugens* feeding

Sl. No	Category (Score)	Genotypes	0 HAR	24 HAI	48 HAI	72 HAI
1	R (3)	Bavdi	2.85 (9.71)*cd	4.35 (12.03)c	5.74(13.86)bc	5.61(13.70)b
2	R (3)	Bacharya Khuta	2.79 (9.61)d	4.38 (12.08)c	5.43(13.47)cd	4.23(11.87)fg
3	HR (1)	Sonahanan	1.55 (7.15)l	4.21 (11.84)c	5.82(13.96)cd	5.64(13.73)de
4	HR (1)	Mata Meher	2.07 (8.28)hi	5.21 (13.19)a	6.30(14.53)a	5.98(14.16)a
5	HR (1)	Hermonona	1.81 (7.73)j	4.34 (12.02)c	5.38(13.41)cd	4.52(12.27)de
6	HR (1)	Manipuri Black	1.56 (7.17)kl	5.10 (13.05)a	6.15(14.36)ab	5.53(13.60)b
7	R (3)	Sati Kh-16	2.19 (8.51)gh	3.32 (10.50)ijk	5.00(12.92)def	4.69(12.51)cd
8	R (3)	Uttar Bangalocal-3	2.39 (8.90)ef	2.98 (9.93)mn	4.09(11.66)lmn	3.88(11.37)hij
9	R (3)	Bandi Marhan	2.35 (8.82)f	2.65 (9.38)q	4.14(11.74)klmn	3.76(11.18)jk
10	R (3)	Chapa khusi-1	2.74 (9.53)d	3.37 (10.57)hij	4.16(11.77)jklmn	4.14(11.74)fg
11	R (3)	Chadai Guda	1.69 (7.46)jk	3.22 (10.34)jkl	4.69(12.51)fgh	4.58(12.36)d
12	R (3)	Manipuri	2.53 (9.15)e	4.74 (12.57)b	4.03(11.58)mn	3.85(11.31)ijk
13	R (3)	Mujni	1.61 (7.28)kl	2.79 (9.61)opq	4.25(11.90)ijklm	4.12(11.70)fgh
14	R (3)	Kakharua	2.38 (8.88)ef	2.90 (9.80)no	4.55(12.32)ghij	4.13(11.73)fg
15	R (3)	Lechade dhan	2.53 (9.16)e	3.12 (10.18)lm	4.80(12.65)efg	4.65 (12.45)cd
16	MR (5)	Rajesh	1.98 (8.09)i	3.75 (11.16)e	4.24(11.89)jklm	4.13(11.73)fg
17	MR (5)	Meghi	3.22 (10.34)a	3.69(11.08)e	4.49 (12.23)ghijk	4.07 (11.64)ghi
18	MR (5)	Balam	3.03 (10.03)b	3.30 (10.46)ijk	4.09(11.66)lmn	3.89(11.37)hij
19	MR (5)	Khuti Dhan	2.74 (9.54)d	3.99 (11.53)d	5.19(13.17)de	4.32(12.00)ef
20	MR (5)	Gerua Mudi	2.80 (9.63)d	3.64 (10.99)ef	4.32(11.99)hijklm	3.86(11.34)ij
21	MR (5)	Sakta-1	1.19 (6.26)m	3.17 (10.25)kl	5.21(13.20)de	4.86(12.73)c
22	MR (5)	Shial Bhomra	1.24 (6.39)m	2.75 (9.55)pq	4.21(11.84)jklm	3.88(11.35)ij

23	MR (5)	Binni	2.14 (8.42)gh	3.58 (10.91)efg	4.22(11.85)jklm	4.01(11.56)ghi
24	MR (5)	Kalshepa Kh-16	2.16 (8.46)gh	3.64 (11.00)ef	4.43(12.14)ghijkl	4.20(11.82)fg
25	MR (5)	Maria Dhan-2	2.80 (9.64)d	3.60 (10.94)efg	4.13(11.72)klmn	3.86(11.33)fg
26	MR (5)	Maha Baisur	1.64 (7.37)kl	4.22 (11.86)c	3.78(11.22)no	3.62(10.97)k
27	MR (5)	Mudi Futa	1.54 (7.13)l	2.90 (9.80)nop	4.65 (12.45)fghi	4.22(11.85)fg
28	MR (5)	Sarka Dhan	1.18 (6.24)m	3.52 (10.82)fgh	4.34(12.03)hijklm	4.23(11.86)fg
29	HR (1)	Salkati (Check)	3.14 (10.20)ab	4.30 (11.96)c	5.95(14.12)ab	4.88(12.77)c
30	HR (1)	PTB-33 (Check)	2.98 (9.95)bc	4.23 (11.87)c	5.16(13.13)de	4.32(12.00)ef
31	HS (9)	TN1 (Check)	2.29 (8.71)fg	3.44 (10.69)ghi	3.62(10.98)o	2.84(9.71)l
DF			30	30	30	30
Treatment Sum of Squares			132.52	94.13	83.08	75.56
F value			681.2918	669.3151	120.0399	289.2772
p-Value			<.0001	<.0001	<.0001	<.0001
CV(%)			0.95	0.620	1.210	0.78
SE(d)			0.066	0.056	0.124	0.076
Tukey HSD at 1%			0.296	0.252	0.558	0.343

Mean of three replications in a column, followed by the common letter are not significantly different (P = 0.05)

*Figures in parenthesis are transformed square root values; HAI: Hour after infestation

HR: Highly Resistant, MR: Moderately Resistant, MS: Moderately Susceptible, HS: Highly Susceptible

Table S4: Polyphenol oxidase (PPO) activity (UA/g FW) in red rice genotypes after *N. lugens* feeding

Sl. No	Category (Score)	Genotypes	0 HAR	24 HAI	48 HAI	72 HAI
1	R (3)	Bavdi	6.28 (14.51)ef	8.53 (16.98)c	11.49 (19.82)bc	8.35 (16.80)abcd
2	R (3)	Bacharya Khuta	6.76 (15.07)cd	8.49 (16.94)c	9.65 (18.10) ef	7.83 (16.25)abcd
3	HR (1)	Sonahanan	7.62 (16.02)b	8.61 (17.07)c	11.87 (20.15)b	9.53 (17.98)ab
4	HR (1)	Mata Meher	7.57 (15.97)b	10.06 (18.49)a	12.36 (20.58)a	9.65 (18.10)ab
5	HR (1)	Hermonona	6.85 (15.17)c	8.37 (16.82)c	9.89 (18.33)e	7.86 (16.28)abcd
6	HR (1)	Manipuri Black	6.34 (14.58)e	8.98 (17.44)b	10.62 (19.02)d	8.90 (17.36)abc
7	R (3)	Sati Kh-16	4.54 (12.30)m	6.72 (15.02)fg	8.36(16.81)jk	7.60 (16.01)bcde
8	R (3)	Uttar Bangalocal-3	5.65 (13.75)i	6.15 (14.36)h	7.85 (16.27)mn	5.85 (14.00)ef
9	R (3)	Bandi Marhan	5.72 (13.83)hi	7.75 (16.16)d	8.90 (17.36)hi	7.33 (15.71)cdef
10	R (3)	Chapa khusi-1	5.89 (14.04)g	7.11 (15.47)e	9.21 (17.67)gh	6.50 (14.78)def
11	R (3)	Chadai Guda	5.82 (13.96)gh	7.00 (15.34)ef	7.30 (15.67)pq	6.47 (14.73)def
12	R (3)	Manipuri	6.37 (14.62)e	7.08 (15.44)e	8.30 (16.74)k	6.88 (15.20)cdef
13	R (3)	Mujni	4.75 (12.59)l	5.70 (13.81)ij	6.54 (14.81)r	5.59(13.67)fg
14	R (3)	Kakharua	5.35 (13.38)j	6.15 (14.36)h	7.93 (16.36)lmn	6.79 (15.11)def
15	R (3)	Lechade dhan	4.65 (12.46)lm	5.98 (14.15)h	8.78 (17.23)i	5.82 (13.96)ef
16	MR (5)	Rajesh	4.99 (12.91)k	6.63 (14.93)g	8.20 (16.64)kl	6.91 (15.24)cdef
17	MR (5)	Meghi	2.33 (08.77)p	5.96 (14.13)hi	7.32 (15.70)pq	6.75 (15.06)def
18	MR (5)	Balam	4.15 (11.76)o	5.62 (13.71)j	7.53 (15.93)op	5.80 (13.94)ef
19	MR (5)	Khuti Dhan	4.75 (12.59)l	6.00 (14.18)h	7.39 (15.77)pq	6.60(14.88)def
20	MR (5)	Gerua Mudi	6.65 (14.94) d	7.52 (15.92)d	8.20 (16.64)kl	7.29 (15.66)def
21	MR (5)	Sakta-1	6.15 (14.36)f	6.54 (14.82)g	9.36 (17.81)fg	7.36(15.74)cdef
22	MR (5)	Shial Bhomra	4.98 (12.89)k	7.65 (16.05)d	9.24 (17.70)gh	6.84 (15.16)def
23	MR (5)	Binni	5.68 (13.79)hi	6.55 (14.83)g	8.30(16.74)kl	6.93 (15.26)cdef

24	MR (5)	Kalshepa Kh-16	4.27 (11.92)o	6.08 (14.27)h	7.75 (16.16)no	6.54 (14.82)def
25	MR (5)	Maria Dhan-2	5.66 (13.76)i	6.21 (14.43)h	8.14 (16.58) klm	6.53 (14.81)def
26	MR (5)	MahaBaisur	4.40 (12.11)n	5.71 (13.82)ij	7.11 (15.47)q	4.24 (11.89)g
27	MR (5)	MudiFuta	5.92 (14.08)g	6.53 (14.80)g	8.64 (17.09)ij	7.25 (15.62)cdef
28	MR (5)	SarkaDhan	4.92 (12.82)k	6.79 (15.11)fg	9.67 (18.12)ef	7.94 (16.37)abcd
29	HR (1)	Salkati (Check)	7.92 (16.35)a	9.16 (17.61)b	11.49 (19.81)c	9.52 (17.97)ab
30	HR (1)	PTB-33 (Check)	6.31 (14.55)e	9.90 (18.34)a	11.35 (19.68)c	9.83 (18.27)a
31	HS (9)	TN1 (Check)	4.61 (12.40)m	5.19 (13.17)k	6.74 (15.05)r	6.88 (15.20)def
DF			30	30	30	30
Treatment Sum of Squares			210.76	184.47	218.05	186.34
F value			3138.22	794.00	845.31	17.46
p-Value			<.0001	<.0001	<.0001	<.0001
CV(%)			0.35	0.57	0.54	3.84
SE(d)			0.039	0.072	0.076	0.487
Tukey HSD at 1%			0.1736	0.3228	0.3402	2.1879

Mean of three replications in a column, followed by the common letter are not significantly different (P = 0.05)

*Figures in parenthesis are transformed square root values; HAI: Hour after infestation

HR: Highly Resistant, MR: Moderately Resistant, MS: Moderately Susceptible, HS: Highly Susceptible

Table S5: Superoxide dismutase (SOD) activity (mol UA/mg protein) in red rice genotypes after *N. lugens* feeding

**Sl. No**	**Category (Score)**	**Genotypes**	**0 HAR**	**24 HAI**	**48 HAI**	**72 HAI**
1	R (3)	Bavdi	4.78(12.63)*a	5.22(13.20)ab	5.33(13.35)a	5.08 (13.02)a
2	R (3)	Bacharya Khuta	5.07(13.01)a	5.30(13.31)ab	5.38(13.42)a	5.03 (12.96)a
3	HR (1)	Sonahanan	4.67(12.48)a	5.62(13.71)a	5.53(13.61)a	4.99 (12.91)a
4	HR (1)	Mata Meher	4.91(12.80) a	5.22(13.20)ab	5.37(13.39)a	5.03 (12.96)a
5	HR (1)	Hermonona	4.73(12.57)a	5.07(13.01)b	5.22(13.20)a	4.98 (12.90)a
6	HR (1)	Manipuri Black	4.96(12.87)a	5.34(13.37)ab	5.37(13.39)a	4.98 (12.90)a
7	R (3)	Sati Kh-16	3.19(10.28)c-g	3.97(11.49)cd	4.10(11.69)bc	4.16 (11.76) b
8	R (3)	Uttar Bangalocal-3	3.39(10.61) b-e	3.60(10.94)def	4.11(11.70)bc	3.83 (11.29)bcd
9	R (3)	Bandi Marhan	3.38(10.60)b-f	3.53(10.83)efg	3.98(11.50)bc	3.79 (11.23)bcd
10	R (3)	Chapa khusi-1	3.37(10.57)b-g	3.64(10.99)def	3.97(11.49)bc	3.67 (11.04)cde
11	R (3)	Chadai Guda	3.23(10.36) b-g	3.44(10.68)f-h	4.08(11.66)bc	3.52 (10.81)def
12	R (3)	Manipuri	3.43(10.68)bcd	3.98(11.50)cd	4.22(11.85)b	4.08 (11.66)bc
13	R (3)	Mujni	3.83(11.29)bc	3.88(11.36)cde	4.41(12.12)b	4.08 (11.66)bc
14	R (3)	Kakharua	2.98(9.93) d-h	3.33(10.52)f-i	4.07(11.63)bc	3.64 (10.99)cde
15	R (3)	Lechade dhan	3.87(11.34) b	4.18(11.80)c	4.40(12.11)b	4.17 (11.78)b
16	MR (5)	Rajesh	2.55(9.18)hi	3.01(10.00)ij	3.23(10.35)efg	2.95 (9.89) h
17	MR (5)	Meghi	2.75 (9.55) ghi	3.04 (10.05)ij	3.23 (10.35)efg	3.10 (10.15) fgh
18	MR (5)	Balam	2.55(9.19)hi	3.14(10.21)g-j	3.30(10.47)d-g	3.27 (10.42)e-h
19	MR (5)	Khuti Dhan	2.90(9.80) d-h	3.30(10.47)f-i	3.52(10.81)de	3.39 (10.60)d-g
20	MR (5)	Gerua Mudi	2.78(9.59)e-i	3.07(10.09)hij	3.50(10.78)de	3.09 (10.12)fgh
21	MR (5)	Sakta-1	2.81(9.64)d-i	2.98(9.95)ij	3.25(10.39)efg	3.30 (10.47)e-h
22	MR (5)	Shial Bhomra	2.75(9.55)f-i	3.08(10.11)hij	3.36(10.56)d-g	3.33 (10.52)e-h

23	MR (5)	Binni	2.26(8.64)ij	2.95(9.89)ij	3.30(10.47)d-g	3.16 (10.24) fgh
24	MR (5)	Kalshepa Kh-16	2.55(9.19)hi	2.95(9.89)ij	3.17(10.25)efg	2.97 (9.93)gh
25	MR (5)	Maria Dhan-2	2.25(8.63) ij	2.92(9.83)j	3.07(10.09)fg	3.07 (10.09)gh
26	MR (5)	MahaBaisur	2.28(8.69)ij	2.52(9.13)k	3.18(10.27)efg	3.09 (10.13) fgh
27	MR (5)	MudiFuta	2.92(9.83)d-h	2.99(9.96)ij	3.69(11.08)cd	3.65 (11.01) cde
28	MR (5)	SarkaDhan	2.76(9.56)e-i	2.98(9.93)ij	3.37(10.57)def	3.32 (10.50)e-h
29	HR (1)	Salkati (Check)	4.74(12.57)a	5.08(13.02)b	5.08(13.02)a	4.97 (12.88)a
30	HR (1)	PTB-33 (Check)	4.88(12.77) a	5.08(13.02) b	5.23(13.22)a	4.83 (12.69)a
31	HS (9)	TN1 (Check)	1.93(7.99)j	2.23(8.59)k	2.97(9.92)g	2.11 (8.35)i
DF			30	30	30	30
Treatment Sum of Squares			203.98	187.09	132.68	132.24
F value			83.33	210.09	138.77	121.42
p-Value			<.0001	<.0001	<.0001	<.0001
CV (%)			2.71	1.55	1.54	1.70
SE(d)			0.233	0.141	0.146	0.156
Tukey HSD at 1%			1.047	0.632	0.654	0.699

Mean of three replications in a column, followed by the common letter are not significantly different (P = 0.05)

*Figures in parenthesis are transformed square root values; HAI: Hour after infestation

HR: Highly Resistant, MR: Moderately Resistant, MS: Moderately Susceptible, HS: Highly Susceptible

Table S6: Catalase (CAT) activity (mol UA/mg protein) in red rice genotypes after *N. lugens* feeding

Sl. No	Category (Score)	Genotypes	0 HAR	24 HAI	48 HAI	72 HAI
1	R (3)	Bavdi	37.14 (37.55)*a	45.55(42.45)a	32.43(34.72)a	19.42(26.15)a
2	R (3)	Bacharya Khuta	32.03 (34.47)b	42.01(40.40)ab	25.42(30.28)b	17.27(24.56)ab
3	HR (1)	Sonahanan	19.50(26.21)ghijk	34.88(36.20)def	22.72(28.46)bcde	12.37(20.59)defg
4	HR (1)	Mata Meher	25.09(30.06)def	40.04(39.26)bc	21.64(27.72)bcdef	14.40(22.30)bcde
5	HR (1)	Hermonona	26.82(31.19)cd	36.58(37.22)cde	21.72(27.78)bcdef	13.65(21.68)bcdef
6	HR (1)	Manipuri Black	23.54(29.02)defg	37.49(37.75)cd	17.27(24.56)ghijklm	14.14(22.09)bcde
7	R (3)	Sati Kh-16	18.50(25.48)ijk	31.42(34.09)fgh	20.65(27.03)cdefg	12.67(20.85)cdefg
8	R (3)	Uttar Bangalocal-3	13.65(21.68)m	20.41(26.86)no	13.66(21.69)no	11.78(20.08)defg
9	R (3)	Bandi Marhan	12.78 (20.95)m	27.06(31.34)ijkl	17.61(24.82)ghijkl	11.26(19.61)efgh
10	R (3)	Chapa khusi-1	21.39(27.55)fghijk	29.20(32.71)ghijk	19.41(26.14)efghij	13.39(21.47)cdef
11	R (3)	Chadai Guda	19.26(26.03)hijk	22.38(28.24)mn	13.66(21.69)no	10.60(19.00)defg
12	R (3)	Manipuri	22.15(28.08)hijk	27.40(31.56)mn	16.96(24.32)no	12.41(20.63)fgh
13	R (3)	Mujni	19.26(26.03)hijk	29.62(32.97)ghij	16.54(24.00)ijklnm	11.36(19.69)efgh
14	R (3)	Kakharua	25.33 (30.22)def	37.03(37.48)cd	24.36(29.58)bc	13.58(21.62)bcdf
15	R (3)	Lechade dhan	18.50(25.48)jk	25.43(30.28)klm	18.44(25.43)fghijk	11.51(19.84)efgh
16	MR (5)	Rajesh	26.31(30.86)de	35.11(36.34)def	16.14(23.69)jklmn	11.51(19.84)efgh
17	MR (5)	Meghi	13.94 (21.92)lm	22.38 (28.24)mn	14.73 (22.57)lmn	12.25 (20.48)defg
18	MR (5)	Balam	21.75 (27.80)fghij	30.35 (33.43)ghi	20.40(26.85)defgh	16.28 (23.80)abc
19	MR (5)	Khuti Dhan	19.26(26.03)efghij	22.38(28.24)hijkl	18.51(25.48)hijklmn	13.40(21.48)defg
20	MR (5)	Gerua Mudi	17.53(24.75)kl	31.41(34.08)fgh	21.56(27.67)bcdef	15.33(23.05)bcd
21	MR (5)	Sakta-1	22.47(28.29)efghi	25.57(30.38)jklm	16.78(24.18)hijklnm	12.25(20.48)defg
22	MR (5)	Shial Bhomra	11.52(19.84)m	24.42(29.61)lm	14.06(22.02)mno	13.26(21.35)cdef

23	MR (5)	Binni	19.26(26.03)hijk	27.38(31.55)hijkl	22.46(28.29)bcde	13.25(21.34)cdef
24	MR (5)	Kalshepa Kh-16	21.47(27.60)fghij	26.33(30.88)ijkl	19.93(26.51)efghi	12.51(20.71)defg
25	MR (5)	Maria Dhan-2	31.27(34.00)cd	40.48(39.51)cde	18.44(25.43)bcdef	11.60(19.91)bcdef
26	MR (5)	Maha Baisur	12.25(20.48)m	27.47(31.61)hijkl	19.26(26.03)hijklmn	12.58(20.77)defg
27	MR (5)	Mudi Futa	12.41(20.62)m	19.50(26.21)no	15.46(23.15)klmn	11.40(19.73)efgh
28	MR (5)	Sarka Dhan	13.42(21.49)m	20.41(26.86)no	16.37(23.87)klmn	9.81(18.25)efgh
29	HR (1)	Salkati (Check)	23.28(28.85)defgh	35.19(36.39)def	24.20(29.47)bcd	12.58(20.77)cdefg
30	HR (1)	PTB-33 (Check)	24.52(29.68)kl	32.42(34.70)fgh	17.36(24.62)bcdef	13.70(21.72)bcd
31	HS (9)	TN1 (Check)	13.39(21.47)m	17.36(24.63)o	11.34(19.68)o	8.41(16.85)h
DF			30	30	30	30
Treatment Sum of Squares			1757.22	1870.37	835.53	288.87
F value			98.36	122.30	51.06	13.92
p-Value			<.0001	<.0001	<.0001	<.0001
CV(%)			2.880	2.170	2.870	3.960
SE(d)			0.630	0.584	0.603	0.679
Tukey HSD at 1%			2.831	2.620	2.703	3.050

Mean of three replications in a column, followed by the common letter are not significantly different (P = 0.05)

*Figures in parenthesis are transformed square root values; HAI: Hour after infestation

HR: Highly Resistant, MR: Moderately Resistant, MS: Moderately Susceptible, HS: Highly Susceptible

Table S7: Total soluble proteins (mg/g FW) in red rice genotypes after *N. lugens* feeding

Sl. No	Category (Score)	Genotypes	0 HAR	24 HAI	48 HAI	72 HAI
1	R (3)	Bavdi	9.35(17.81)e-l	7.33(15.71)i-l	6.17(14.38)fgh	5.83 (13.98)d-g
2	R (3)	Bacharya Khuta	7.44(15.83)kl	7.17(15.53)jkl	6.17(14.38)fgh	5.33(13.35)fg
3	HR (1)	Sonahanan	8.58(17.04)g-l	7.92(16.34)g-l	6.92(15.25)e-h	5.58(13.67)d-g
4	HR (1)	Mata Meher	7.25(15.62)kl	6.33(14.58)l	5.26(13.26)c-f	4.75(12.59)g
5	HR (1)	Hermonona	8.00(16.43)jkl	7.65(16.06)hijkl	6.75(15.06)e-h	5.25(13.25)d-g
6	HR (1)	Manipuri Black	6.67(14.96)l	6.33(14.58)l	5.92(14.08)fgh	4.83(12.70)g
7	R (3)	Sati Kh-16	8.92(17.37)f-l	8.17(16.61)g-l	8.33(16.78)c-f	8.58(17.04)a-f
8	R (3)	Uttar Bangalocal-3	11.83(20.12)b-i	9.83(18.28)e-k	7.67(16.07)b-h	5.83(13.98)d-g
9	R (3)	Bandi Marhan	12.17(20.41)b-h	11.00(19.37)c-h	9.33(17.79)b-e	7.33(15.71)b-g
10	R (3)	Chapa khusi-1	13.17(21.28)b-e	13.33(21.42)a-e	10.58(18.98)a-d	9.34(17.80)a-d
11	R (3)	Chadai Guda	9.34(17.80)e-l	7.26(15.63)i-l	5.92(14.08)f-h	5.40(13.44)f-g
12	R (3)	Manipuri	8.67(17.12)f-l	7.33(15.71)i-l	5.42(13.46)gh	5.42(13.46)fg
13	R (3)	Mujni	8.08(16.52)jkl	7.25(15.62)i-l	7.17(15.53)e-h	5.48(13.54)efg
14	R (3)	Kakharua	13.42(21.49)abc	12.42(20.63)abc	10.42(18.83)ab	8.67(17.12)a-e
15	R (3)	Lechade dhan	10.67(19.06)d-k	10.83(19.22)d-i	9.42(17.87)b-e	7.58(15.98)b-g
16	MR (5)	Rajesh	13.33(21.42)b-e	11.25(19.60)b-h	9.43(17.89)b-e	7.33(15.71)b-g
17	MR (5)	Meghi	18.47 (25.46)a	16.50 (23.97)a	13.33 (21.42)a	12.17 (20.41)a
18	MR (5)	Balam	15.83(23.45)ab	15.42 (23.12)ab	13.25(21.35)a	11.17 (19.52)ab
19	MR (5)	Khuti Dhan	15.17(22.92)abc	15.17(22.92)abc	11.83(20.12)ab	9.25(17.71)a-e
20	MR (5)	Gerua Mudi	13.17(21.28)b-e	12.08(20.34)a-f	9.42(17.87)b-e	7.25(15.62)b-g
21	MR (5)	Sakta-1	13.92(21.90)a-d	10.83(19.22)d-i	9.25(17.71)b-e	6.92(15.25)c-g

22	MR (5)	Shial Bhomra	11.33(19.67)c-j	9.42(17.87)e-l	8.25(16.69)c-g	7.13(15.49)b-g
23	MR (5)	Binni	10.33(18.75)d-k	10.33(18.75)d-j	8.00(16.43)c-h	6.92(15.25)c-g
24	MR (5)	Kalshepa Kh-16	15.17(22.92)f-l	14.00(21.97)g-l	12.50(20.70)c-f	10.83(19.22)a-f
25	MR (5)	Maria Dhan-2	14.42(22.31)a-d	12.83(20.99)a-f	11.00(19.37)abc	9.25(17.71)a-e
26	MR (5)	Maha Baisur	12.50(20.70)b-f	11.67(19.97)b-g	9.33(17.79)b-e	7.75(16.16)b-g
27	MR (5)	Mudi Futa	12.32(20.55)b-g	10.83(19.22)d-i	9.42(17.87)b-e	8.08(16.52)a-g
28	MR (5)	Sarka Dhan	11.25(19.60)c-j	9.25(17.71)f-l	7.42(15.80)d-h	6.67(14.96)d-g
29	HR (1)	Salkati (Check)	8.42(16.86)h-l	6.32(14.56)l	6.00(14.18)fgh	5.42(13.46)fg
30	HR (1)	PTB-33 (Check)	8.17(16.61)jkl	6.58(14.87)kl	6.08(14.28)fgh	5.50(13.56)efg
31	HS (9)	TN1 (Check)	15.25(22.99)abc	13.17(21.28)a-f	10.67(19.06)b-e	9.25(17.71)b-g
DF			30	30	30	30
Treatment Sum of Squares			672.83	705.85	506.14	408.49
F value			22.84	23.65	20.91	10.22
p-Value			<.0001	<.0001	<.0001	<.0001
CV (%)			5.070	5.410	5.32	7.440
SE(d)			0.809	0.814	0.733	0.94
Tukey HSD at 1%			3.634	3.658	3.294	4.23

Mean of three replications in a column, followed by the common letter are not significantly different (P = 0.05)

*Figures in parenthesis are transformed square root values; HAI: Hour after infestation

HR: Highly Resistant, MR: Moderately Resistant, MS: Moderately Susceptible, HS: Highly Susceptible

Table S8: Crude silica (%) in red rice genotypes after *N. lugens* feeding

Sl. No	Category (Score)	Genotypes	Silica (%)
1	R (3)	Bavdi	4.17 (11.78) b-j
2	R (3)	Bacharya Khuta	4.45 (12.18) b-j
3	HR (1)	Sonahanan	7.25 (15.62) abc
4	HR (1)	Mata Meher	8.42 (16.86) a
5	HR (1)	Hermonona	6.58 (14.87) a-e
6	HR (1)	Manipuri Black	8.08 (16.52) a
7	R (3)	Sati Kh-16	5.58 (13.67) a-h
8	R (3)	Uttar Bangalocal-3	6.42 (14.67) a-f
9	R (3)	Bandi Marhan	4.34 (12.02) b-j
10	R (3)	Chapa khusi-1	5.92 (14.08) a-h
11	R (3)	Chadai Guda	6.36 (14.61) a-g
12	R (3)	Manipuri	6.33 (14.58)a-g
13	R (3)	Mujni	5.25 (13.25)a-i
14	R (3)	Kakharua	5.08 (13.03) a-j
15	R (3)	Lechade dhan	5.92 (14.08) a-h
16	MR (5)	Rajesh	3.31 (10.48) h-j
17	MR (5)	Meghi	3.73 (11.14) d-j
18	MR (5)	Balam	3.19 (10.30) h-j
19	MR (5)	Khuti Dhan	4.00 (11.54) c-j
20	MR (5)	Gerua Mudi	3.92 (11.41) d-j

21	MR (5)	Sakta-1	3.33 (10.52) g-j
22	MR (5)	Shial Bhomra	3.42 (10.65) f-j
23	MR (5)	Binni	3.30 (10.47) h-j
24	MR (5)	Kalshepa Kh-16	3.42 (10.65) f-j
25	MR (5)	Maria Dhan-2	6.50 (14.77)a-e
26	MR (5)	Maha Baisur	3.39 (10.61) f-j
27	MR (5)	Mudi Futa	3.63 (10.99) e-j
28	MR (5)	Sarka Dhan	2.64 (9.36) ij
29	HR (1)	Salkati (Check)	7.42 (15.80) ab
30	HR (1)	PTB-33 (Check)	6.92 (15.25)a-d
31	HS (9)	TN1 (Check)	2.48 (9.06) j
DF			30
Sum of Squares			454.35
F value			12.00
p-Value			<.0001
CV (%)			8.850
SE(d)			0.971
Tukey HSD at 1%			4.119

Mean of three replications in a column, followed by the common letter are not significantly different (P = 0.05)

*Figures in parenthesis are transformed square root values; HAI: Hour after infestation

HR: Highly Resistant, MR: Moderately Resistant, MS: Moderately Susceptible, HS: Highly Susceptible
